# Supplementary material for: Lichenysin-like Polypeptide Production by Bacillus licheniformis B3-15 and Its Antiadhesive and Antibiofilm Properties
Source: Microorganisms. 2023 Jul 20;11(7):1842. doi: 10.3390/microorganisms11071842 (PMC10384595; doi:10.3390/microorganisms11071842)
Supplement: Supplementary file 1 [file microorganisms-11-01842-s001.zip › microorganisms-2440088-supplementary.pdf]

Supplementary materials

Lichenysin-like Polypeptide Production by *Bacillus licheniformis* B3-15 and Its Antiadhesive and Antibiofilm Properties

Vincenzo Zammuto [1,2,3], Maria Giovanna Rizzo [1,*], Claudia De Pasquale [4], Guido Ferlazzo [5,6], Maria Teresa Caccamo [2,7], Salvatore Magazù [2,3,7], Salvatore Pietro Paolo Guglielmino [1] and Concetta Gugliandolo [1,2,*]

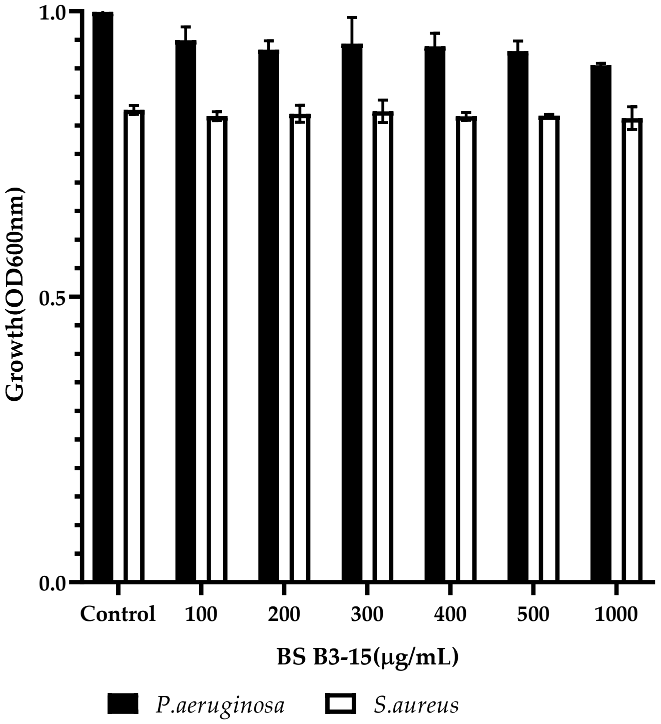

Figure S1. Effects of EPS B3-15 addition at different concentrations (from 100 to 1000 μg/mL) on the growth (OD600nm) of *P. aeruginosa* (a) and *S. aureus* (b). Data represent mean ± SD for six replicates (n = 6).

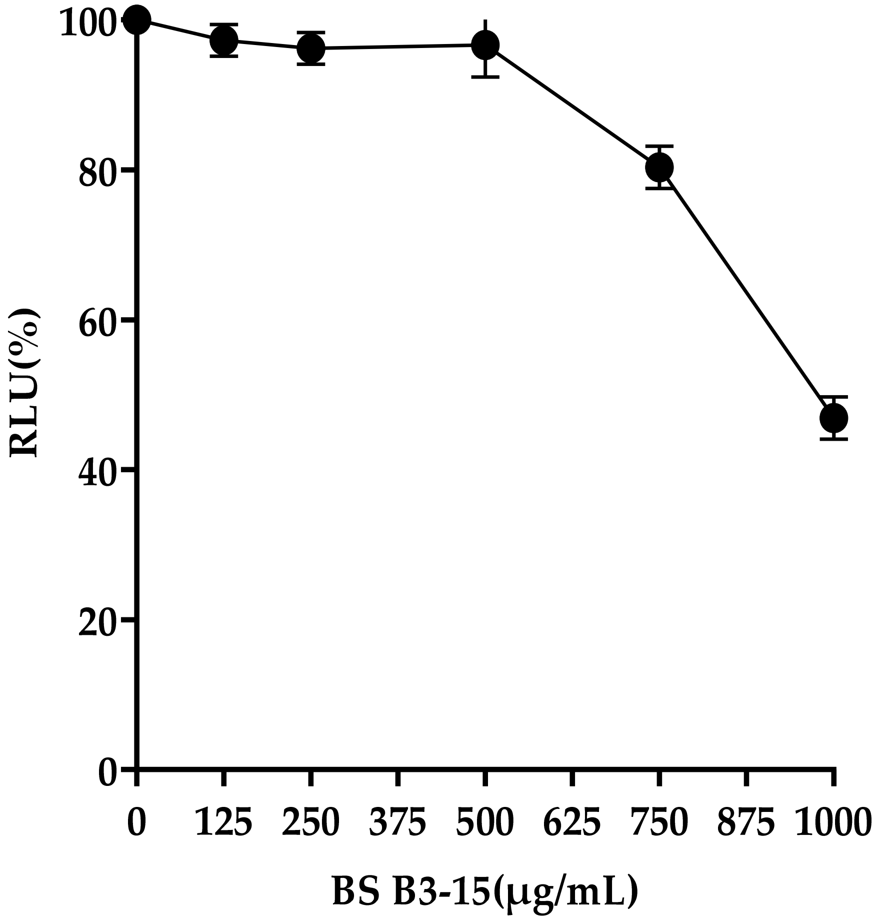

Figure S2. Effects of the BS B3-15 presence at different concentrations on the luminescence of *V. harveyi* G5 after 15 min, as percentage of relative luminescence unit (RLU). Data represent mean ± SD for six replicates (n = 6).
